# Comparative Effectiveness of Secondary Furlow and Buccal Myomucosal Flap Lengthening to Treat Velopharyngeal Insufficiency

**DOI:** 10.1097/GOX.0000000000005375

**Published:** 2023-11-03

**Authors:** Thomas J. Sitzman, Jamie L. Perry, Taylor D. Snodgrass, M’hamed Temkit, Davinder J. Singh, Jessica L. Williams

**Affiliations:** From * Phoenix Children’s Center for Cleft and Craniofacial Care a Division of Plastic Surgery, Phoenix Children’s Hospital, Phoenix, Ariz.; † Division of Plastic Surgery, Mayo Clinic Arizona, Scottsdale, Ariz.; ‡ Department of Communication Sciences and Disorders East Carolina University, Greenville, N.C.; § Department of Clinical Research, Phoenix Children’s Hospital, Phoenix, Ariz.; ¶ Program of Speech and Hearing Science, College of Health Solutions, Arizona State University, Tempe, Ariz.

## Abstract

**Background:**

Secondary Furlow (Furlow) and buccal myomucosal flaps (BMMF) treat velopharyngeal insufficiency by lengthening the palate and retropositioning the levator veli palatini muscles. The criteria for choosing one operation over the other remain unclear.

**Methods:**

A single-center retrospective cohort study was conducted. Thirty-two patients with nonsyndromic, repaired cleft palate were included. All patients underwent a Furlow or BMMF. Outcome measures included (1) resolution of hypernasality 12 months postoperatively, (2) degree of improvement of hypernasality severity; and (3) change in velar length, as measured on magnetic resonance imaging scans obtained preoperatively and 12 months postoperatively. All measures were performed by raters blinded to participants’ medical and surgical history.

**Results:**

Hypernasality was corrected to normal in 80% of the Furlow group and in 56% of the BMMF group. Patients receiving BMMF had more severe hypernasality during preoperative speech evaluation. Both groups had a median decrease of two scalar rating points for severity of hypernasality (*P* = 0.58). On postoperative magnetic resonance imaging, patients who underwent Furlow had a median increased velar length of 6.9 mm. Patients who received BMMF had a median increased velar length of 7.5 mm. There was no statistically significant difference between groups regarding increase in velar length (*P* = 0.95).

**Conclusions:**

Furlow and BMMF procedures increase velar length with favorable speech outcomes. The same degree of improvement for hypernasality was observed across groups, likely explained by the similar increase in velar length achieved. Anatomic changes in palate length and levator veli palatini retropositioning persist 1 year after surgery.

**Takeaways**

**Question:** What is the comparative effectiveness of secondary Furlow and buccal myomucosal flaps in lengthening the velum to treat velopharyngeal insufficiency?

**Findings:** On postoperative magnetic resonance imaging, a secondary Furlow lengthens the velum by a median of 6.9 mm, and buccal myomucosal flaps lengthen the velum by a median of 7.5 mm. There was no significant difference between procedures for increasing the length of the velum (*P* = 0.95).

**Meaning:** Both secondary Furlow and buccal myomucosal flap procedures increase velar length. Anatomic changes in palate length and levator veli palatini retropositioning persist 1 year after surgery.

## INTRODUCTION

The success of primary palate repair is measured by the presence or absence of velopharyngeal insufficiency (VPI).^1–4^ Symptoms of VPI include hypernasal sounding speech and audible nasal emission. Treatment of VPI requires additional surgery to aid the palate in achieving closure with the posterior pharyngeal wall during speech. Palate lengthening procedures are being offered by many surgeons to treat VPI in patients with nonsyndromic cleft palate as they maintain normal functioning of the velopharyngeal port^5^ and have a low risk of obstructive sleep apnea.^6^

Palate lengthening procedures, including secondary Furlow double-opposing Z-plasty (Furlow) and buccal myomucosal flaps (BMMF), are reported to directly address the underlying anatomical concern causing VPI by lengthening the palate and retropositioning the levator veli palatini muscles.^7,8^ Although the goal of both operations is to lengthen the palate, the criteria for choosing one operation over the other remain unclear.^9^ Moreover, although increased velar length is evident during both procedures, there is limited evidence that this lengthening is maintained postoperatively.^10–12^

Studies evaluating the effectiveness of palatal lengthening procedures report improvement of VPI symptoms in most patients, with complete VPI resolution ranging from 37%–69% of patients who received a Furlow^6^ and 50%–83% of patients who underwent BMMF.^8,13–16^ In the BMMF procedure, a full-thickness transverse incision is made just posterior to the hard palate, abnormal muscle attachments to the hard palate release, and then the soft palate is moved posteriorly; the resultant defect between the hard and soft palates is then filled with bilateral flaps of buccal mucosa and buccinator muscle, which are raised with their base in the retromolar trigone and rotated into the palate, using one flap to repair the nasal mucosa and a second to repair the oral mucosa.^8,14^ As BMMFs are designed with a width of 10–15 mm, and this degree of lengthening is usually observed intraoperatively after flap inset to the palate,^9^ our team developed a clinical pathway where BMMFs were used to treat medium to large velopharyngeal gaps during phonation, while patients who presented with small to medium gaps, determined by measures of gap size from a clinical velopharyngeal MRI study, were treated using a secondary Furlow.^17,18^ **(See figure, Supplemental Digital Content 1,** which displays the clinical pathway using gap size to determine re-repair technique for patients with nonsyndromic cleft palate presenting with VPI following primary palate repair. http://links.lww.com/PRSGO/C841.) The purpose of this study was to compare the anatomical change in velar length and the associated speech outcomes of this clinical pathway.

## METHODS

### Subjects

Following approval from our institutional review board, a retrospective review of patients undergoing VPI evaluation at Phoenix Children’s Hospital (Phoenix, Arizona) was conducted. Patients meeting all the following criteria were included: (1) history of nonsyndromic cleft palate, with or without cleft lip, repaired in infancy; (2) presence of hypernasality and/or audible nasal emission on preoperative speech evaluation; and (3) VPI surgically treated using secondary Furlow double-opposing Z-plasty or buccal myomucosal flaps between July 2018 and June 2021. Patients were excluded if they had previously undergone a surgery to treat VPI.

### Clinical Speech Evaluation

Patients were seen by one of three cleft team speech-language pathologists pre- and postoperatively for a clinical visit during which they completed a speech evaluation. Patients’ guardians provided standard consent for clinical treatment, which included obtaining audio recordings. The perceptual speech evaluation included an audio-recorded speech sample with 2–3 minutes of elicited conversation, counting, and a sentence or phrase repetition task using the American English Sentence Sample or American English Phrase Sample as a part of the Cleft Audit Protocol for Speech-Augmented-Americleft Modification (CAPS-A-AM) rating scale.^19^ Recordings were collected in private clinic rooms using a laptop with a plug and play Logitech for Creators Blue Snowball Microphone placed on a table within 12 inches of the patient.

All postoperative speech evaluations for this study were obtained at least 5 months after surgery but no more than 14 months after surgery. If a patient received multiple evaluations during this time frame, the speech evaluation farthest from surgery was used for analysis.

### Speech Reliability Ratings

All recordings were made during a clinical evaluation at least 6 months before being re-rated. The play order of the samples was randomized using the randomize tool in Microsoft Excel.^20^ All archived speech samples were rated by a single speech language pathologist with more than 9 years of experience assessing VPI and trained on the use of the CAPS-A-AM rating scale.^21^ The rater was blinded to subjects’ medical and surgical histories, including whether the sample was obtained pre- or postoperatively. A subset of samples (36%) were re-rated to determine intrarater reliability. The CAPS-A-AM rating scale was used to rate the severity of speech parameters related to VPI, including hypernasality and audible nasal emission.^21^ Hypernasality was rated on a five-point ordinal scale: none, minimal, mild, moderate, severe. Audible nasal emission was rated on a three-point ordinal scale: absent, occasionally present, frequently present.

The MacKay-Kummer Simplified Nasometric Assessment Procedures Test-Revised (SNAP-R)^22,23^ was completed to supplement the perceptual speech evaluation. The SNAP-R provides a number from 0 to 100, called nasalance, that is related to the perception of nasality in speech. Picture-cued subtests were administered to obtain scores for four oral passages and one nasal passage. For oral passages, scores of 22 or more are indicative of hypernasality. For the nasal passage, scores 45 or less are indicative of hyponasality.

### Nasopharyngoscopy

Nasopharyngoscopy was completed preoperatively by using a 2.4-mm Pentax flexible fiber nasopharyngolaryngoscope that was inserted with sterile lubricant into either the right or left naris. The speech sample obtained varied across patients based on the oral pressure consonants each patient could accurately articulate as identified by a team speech language pathologist.

For this study, all archived nasopharyngoscopy recordings were randomized and rated by a single trained speech-language pathologist who was blinded to subjects’ medical and surgical histories. Closure was measured as the total percentage closure of the velopharyngeal port (0%–100%) using standardized reporting criteria.^24^

### Magnetic Resonance Imaging

**Fig. 1.**
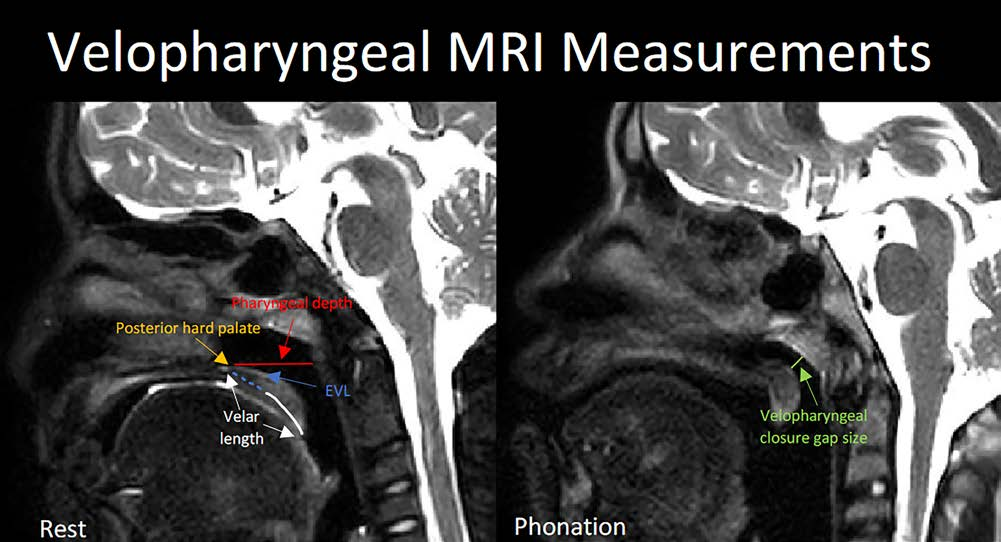
Velopharyngeal MRI measures at rest of pharyngeal depth in red, effective velar length (EVL) in blue, velar length in white, and velopharyngeal gap size during phonation in green. The posterior hard palate is identified in yellow.

Patients completed a whole head MRI on a 3T Phillips scanner using a fully awake, nonsedated, noncontrast protocol. The imaging protocol included a high-resolution T2-weighted turbo-spin-echo 3D sequence obtained at rest, followed by sagittal and oblique coronal T2 images obtained during sustained/i/ (“eee”) phonation. A rater with over 15 years of experience in MRI evaluations of velopharyngeal anatomy conducted the measures on MRI data. The rater was not involved in the direct care of any of the patients. Continuous variables measured in millimeters included (1) velar length-distance from the posterior nasal spine to the tip of the uvula; (2) effective velar length-linear distance from the posterior border of the hard palate to the point of levator muscle insertion into the velum; (3) pharyngeal depth-posterior nasal spine to the posterior pharyngeal wall; (4) effective velopharyngeal ratio-effective velar length divided by pharyngeal depth; and (5) velopharyngeal closure gap size during sustained phonation.^25^ Figure 1 outlines velopharyngeal MRI measures taken.

### Surgical Selection

Surgery selection was completed after the preoperative speech evaluation and once imaging of the velopharynx was obtained. Imaging was reviewed jointly by the treating surgeon and speech-language pathologist. Patients who presented with a small or medium (<5 mm) velopharyngeal gap during phonation received a Furlow while patients presenting with a medium to large gap (≥5 mm) received BMMF. The Furlow procedure was performed as described by Chen et al,^18^ and the BMMF procedure was performed as described by Elsherbiny et al.^26^

### Analysis

The data were summarized using frequencies and proportions for categorical variables and using median and interquartile range (IQR) for continuous variables. Comparisons between groups were conducted using the Wilcoxon rank-sum test for continuous variables and the chi-square test for categorical variables. The significance level was set at 0.05. Intrarater reliability for speech ratings was calculated using a linear weighted Gwet’s AC2 statistic. This statistic was chosen because of the asymmetrical distribution of hypernasality severity ratings and audible nasal emission ratings. A Gwet’s AC2 provides a coefficient of reliability like other reliability statistics. The kappa statistic was not used to avoid the kappa paradox of high rater agreement but low reliability.^27,28^ Statistical analyses were performed using the statistical software package SAS 9.4 (SAS Institute, Cary, N.C.).

## RESULTS

A total of 32 patients met the inclusion criteria: 12 patients received a secondary Furlow, and 20 patients received BMMF to treat VPI. A full description of patient demographics by surgery type is reported in Table 1. At the time of preoperative speech evaluation, patients in the Furlow group were a median age of 6.8 years and patients in the BMMF group were a median age of 5 years. There was no statistically significant difference in age distribution between groups (*P* = 0.53). There was no statistically significant difference between groups for gender, race, ethnicity, cleft palate type, primary palate technique or presence of Pierre Robin sequence. All patients in both groups were English speaking.

**Table 1.** Patient Demographics

	Secondary Furlow (N = 12), n (%)	Buccal Flap Lengthening (N = 20), n (%)	*P*
Sex			0.29
Male	8 (67)	9 (45)	
Female	4 (33)	11 (55)	
Race			0.13
White	10 (83)	20 (100)	
American Indian/Alaska Native	1 (8)	0 (0)	
Asian	1 (8)	0 (0)	
Ethnicity			0.27
Hispanic or Latino	3 (25)	10 (50)	
Non-Hispanic or Latino	9 (75)	10 (50)	
Age (median (q1, q3))	6.8 (5, 8.8)	5 (4, 9.8)	0.53
Cleft palate type			0.27
Veau I	0 (0)	3 (15)	
Veau II	3 (25)	6 (30)	
Veau III	6 (50)	5 (25)	
Veau IV	2 (17)	6 (30)	
Unknown Veau type	1 (8)	0 (0)	
Primary palate repair technique			0.16
Straight line with IVVP	8 (67)	9 (45)	
Furlow Z-plasty	1 (8)	8 (40)	
Unknown	3 (25)	3 (15)	
Pierre Robin sequence	0 (0)	5 (25)	0.13
Surgeon treating VPI			0.07
Surgeon A	8 (67)	6 (30)	
Surgeon B	4 (33)	13 (65)	
Surgeon C	0 (0)	1 (5)	

## PREOPERATIVE FINDINGS

### Speech Findings

Intrarater reliability (AC2) was 0.82 (95% CI 0.74–0.90) for hypernasality and 0.57 (95% CI 0.41–0.73) for audible nasal emission. Percentage agreement for ratings from recordings was 91% for hypernasality and 80% for audible nasal emission.

Approximately half (55%) of the patients in the BMMF group presented with severe hypernasality, whereas only one patient (8%) in the Furlow group was rated as severe. There was a statistically significant difference in hypernasality ratings between the two groups (*P* = 0.03) with the patients in the BMMF group having more severe ratings than the Furlow group. Nasometry scores were consistent with the hypernasality ratings: nasometry scores for oral passages were significantly higher in patients in the BMMF group, compared with patients in the Furlow group.

### Nasopharyngoscopy Findings

Due to the COVID-19 pandemic and a hospital policy that paused aerosolizing procedures, not all patients completed preoperative nasopharyngoscopy. Four of the 12 patients in the Furlow group (33%) and nine of 20 patients in the BMMF group (45%) completed nasopharyngoscopy. On nasopharyngoscopy, a statistically significant difference was found between groups for total closure of the velopharyngeal port (*P* = 0.01), with the Furlow group having a higher percentage of closure (85%) compared with the BMMF group (60%).

### MRI Findings

All patients (N = 32) underwent preoperative MRI. On preoperative MRI, there was no significant difference between groups for velar length (*P* = 0.90), effective velar length (*P* = 0.62), pharyngeal depth (*P* = 0.11) or effective velopharyngeal ratio (*P* = 0.43). The BMMF group presented with a larger median velopharyngeal closure gap during sustained phonation of 6.2mm as compared with that of the Furlow group of 2.3mm (*P* = 0.03).

Ratings for total velopharyngeal closure on nasopharyngoscopy and velopharyngeal closure gap on MRI are consistent with the more severe speech ratings and higher nasometry scores in the BMMF group. Although both groups presented with similar velopharyngeal anatomy at rest, the BMMF group presented with a larger gap size during phonation on both nasopharyngoscopy and MRI. The larger gap size during phonation in the BMMF group may be attributed to limited elevation of the velum that was observed across several patients.

A full summary of preoperative speech and imaging findings is reported in Table 2.

**Table 2.** Preoperative Speech and Imaging Findings

	Secondary Furlow, n (%)	Buccal Flap Lengthening n (%)	*P*
Hypernasality*	N = 12	N = 20	0.03†
None	0 (0)	0 (0)	
Minimal	1 (8)	0 (0)	
Mild	3 (25)	4 (20)	
Moderate	7 (58)	5 (25)	
Severe	1 (8)	11 (55)	
Audible nasal emission*			0.28
None	3 (25)	9 (45)	
Occasional	1 (8)	4 (20)	
Frequent	8 (67)	7 (35)	
	Median (q1, q3)	Median (q1, q3)	*P*
Nasometry‡	N = 12	N = 20	
* Oral passages*			
Bilabial plosives	28 (20, 40)	39 (34, 44)	0.08
Lingual-alveolar plosives	29 (21, 45)	43 (35, 55)	0.04†
Velar plosives	29 (22, 39)	42 (37, 49)	0.02†
Sibilant fricatives	36 (23, 47)	50 (41, 55)	0.03†
* Nasal passage*	58 (49, 64)	63 (56, 67)	0.31
Nasopharyngoscopy	N = 4	N = 9	
VP§ percentage closure¶	85 (80, 88)	60 (30, 70)	0.01†
MRI	N = 12	N = 20	
* Rest measures*			
Velar length (mm)	20.1 (18.6, 24.2)	20.6 (17.8, 24)	0.90
Effective velar length (mm)	8.8 (7.3, 9.2)	9.3 (6.4, 11.3)	0.62
Pharyngeal depth (mm)	17.8 (14.8, 18.2)	20.3 (19.2, 25.1)	0.11
Effective VP§ ratio	0.5 (0.4, 0.5)	0.4 (0.3, 0.5)	0.43
* Phonation of/i/*			
VP§ gap (mm)	2.3 (2.0, 2.7)	6.2 (3.5, 8.7)	0.03‡

* Ratings assigned by blinded review of speech sample recording obtained at patients’ preoperative evaluation.

† Indicates a statistically significant difference between groups.

‡ Picture-cued subtests.

§ Velopharyngeal.

¶ Total percentage closure assigned by blinded review of video recorded nasopharyngoscopy examinations.

## POSTOPERATIVE CHANGES

### Changes in Speech

The median time from surgery to the postoperative speech evaluation was 9.5 (5.9–11.3) months for the Furlow group and 11.5 (7.1–12.1) months for the BMMF group.

Both groups achieved the same median decrease in hypernasality of two scalar rating points. Hypernasality was corrected to normal in 80% of the patients in the Furlow group and 56% of the BMMF group. The Furlow group had a median decrease in audible nasal emission of one scalar rating point and the BMMF group, a decrease of 0.5. Audible nasal emission was corrected to normal in 60% of the Furlow group and 50% of the BMMF group. Audible nasal emission was worse for 39% of the BMMF group, most frequently moving from “none” to “occasionally present.”

There was no significant difference in the change in hypernasality (*P* = 0.58), audible nasal emission (*P* = 0.63), or nasometry scores between patients who had a Furlow and patients who received BMMF.

### Anatomic Changes Using Postoperative MRI

Of the 32 patients, a subset of 15 patients had both a pre- and postoperative MRI to analyze the change in velopharyngeal anatomy. The median time from surgery to postoperative MRI was 15.4 (11.2–25.6) months for the Furlow group and 9.7 (8.6–12.6) months for the BMMF group (*P* = 0.15).

**Fig. 2.**
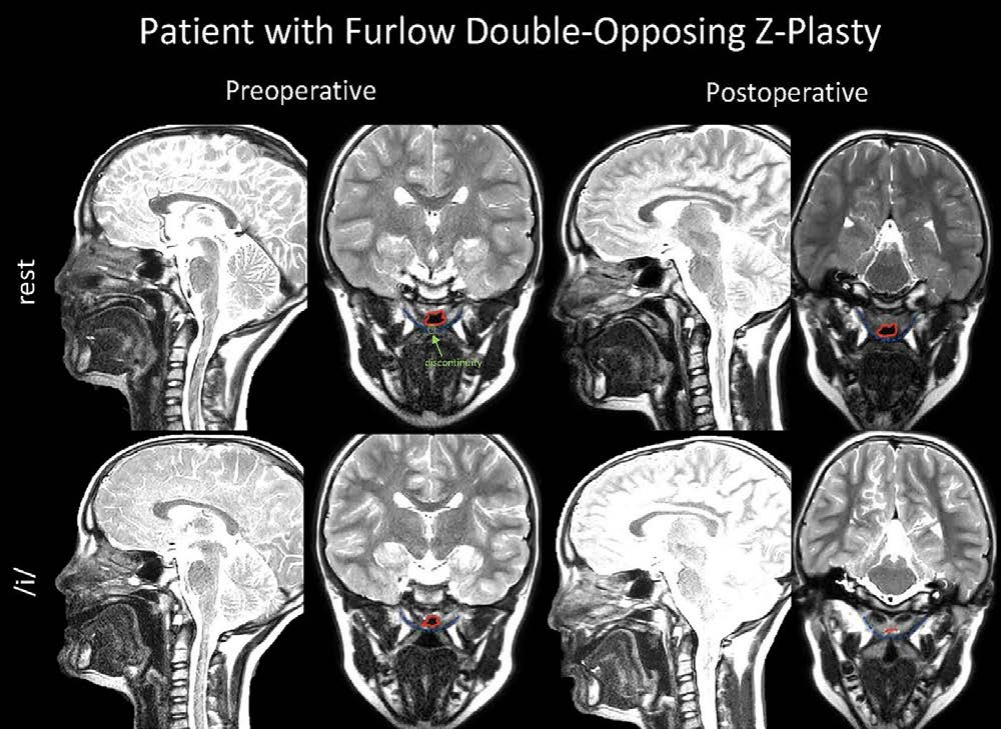
Preoperative (left) and postoperative (right) MRI images of a patient who underwent Furlow double-opposing Z-plasty. Images are taken at rest (top) and during sustained phonation of/i/ (bottom) in the sagittal and oblique coronal views. The velopharyngeal port is outlined in red. The levator veli palatini is outlined in blue. Levator discontinuity is shown in green.

Patients in the Furlow group had a median increase in velar length of 6.9 mm and a median increase in effective velar length of 6.3 mm (Fig. 2). Due to the increase in velar length, the effective velopharyngeal ratio was also increased. The median gap size during phonation decreased by 2 mm.

**Fig. 3.**
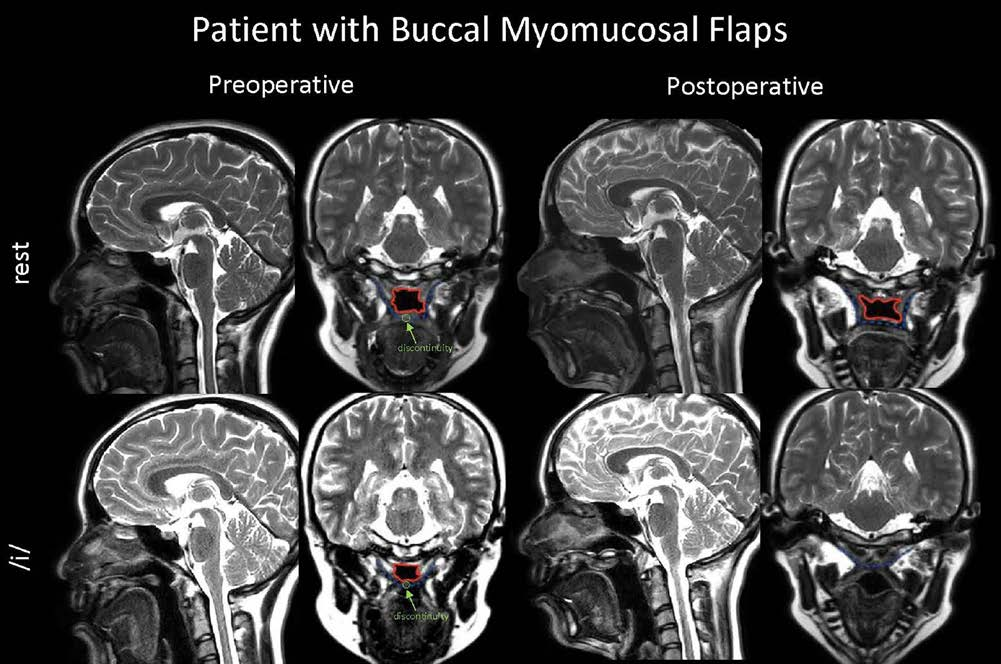
Preoperative (left) and postoperative (right) MRI images of a patient who underwent buccal myomucosal flaps. Images are taken at rest (top) and during sustained phonation of/i/ (bottom) in the sagittal and oblique coronal views. The velopharyngeal port is outlined in red. The levator veli palatini is outlined in blue. Levator discontinuity is shown in green.

Patients in the BMMF group had a median increase in velar length of 7.5 mm and a median increase in effective velar length of 4.4 mm (Fig. 3) The effective velopharyngeal ratio was also increased in the BMMF group. The median gap size during phonation decreased by 5 mm.

There was no statistically significant difference between patients who underwent Furlow versus BMMF in the increase of velar length (*P* = 0.95), effective velar length (*P* = 0.36), or effective velopharyngeal ratio (*P* = 0.43), nor was there a difference between groups in the decrease in gap size (*P* = 0.08).

A full summary of postoperative speech and anatomic changes is reported in Table 3.

**Table 3.** Postoperative Speech and Anatomic Changes

	Secondary Furlow, n (%)	Buccal Flap Lengthening, n (%)	*P*
Resolution of hypernasality	N = 10†	N = 18*	
Corrected to normal (none/minimal)	8 (80)	10 (56)	0.37
Better	1 (10)	6 (33)	
Unchanged	1 (10)	2 (11)	
Worse	0 (0)	0 (0)	
Resolution of audible nasal emission			0.28
Corrected to normal (none)	6 (60)	9 (50)	
Better	1 (10)	2 (11)	
Unchanged	1 (10)	0 (0)	
Worse	1 (10)	7 (39)	
	Median (q1, q3)	Median (q1, q3)	*P*
Change in VPI symptoms			
Change in hypernasality	−2 (−2, −1)	−2 (−3, −1)	0.58
Change in audible nasal emission	−1 (−1.25, −0.75)	−0.5 (−1.75, 0)	0.63
Change in nasometry			
Oral passages			
Bilabial plosives	−10 (−18, −5)	−22 (−28, 1)	0.85
Lingual-alveolar plosives	−8 (−18, −4)	−11 (−40, 0)	0.71
Velar plosives	−9 (−12, −5)	−10 (−32, 3)	0.89
Sibilant fricatives	−14 (−25, −5)	−14 (−40, 5)	0.87
Nasal passage	3 (−4, 4)	−1.5 (−12, 6)	0.61
Anatomic changes (MRI)	N = 5	N = 10	
* Rest measures*			
Velar length (mm)	+6.9 (6.9, 7.9)	+7.5 (5.6, 10.6)	0.95
Effective velar length (mm)	+6.3 (4.5, 6.9)	+4.4 (3.6, 8.5)	0.36
Effective VP ratio	+0.4 (0.3, 0.4)	+0.3 (0.1, 0.4)	0.43
* Phonation of/i/*			
VP gap (mm)	−2.0 (−2.3, −1.0)	−5.0 (−6.2, −2.3)	0.08

* Two patients in each group did not complete a postoperative speech evaluation, and one patient in the Furlow group did not complete the GFTA as a part of the postoperative evaluation.

## DISCUSSION

**Fig. 4.**
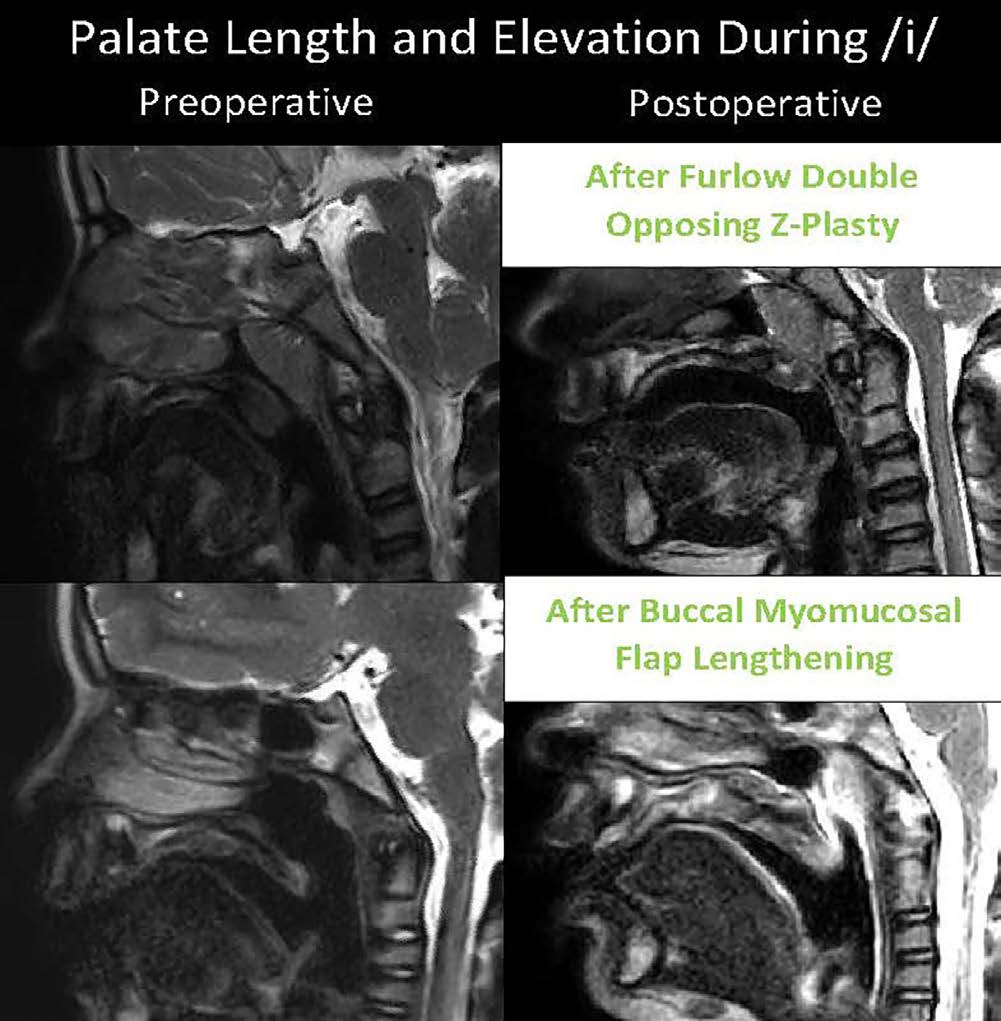
Preoperative and postoperative MRIs of a patient who underwent a secondary Furlow double-opposing Z-plasty (top) and a patient who underwent buccal myomucosal flaps (bottom) in the sagittal plane. Images highlight the difference in preoperative velopharyngeal gap size, velar length gained, and elevation of the velum during sustained/i/ between patients.

Furlow double-opposing Z-plasty and buccal myomucosal flaps are both effective surgical options to lengthen the palate.^8,29^ In the current study, patients in both the Furlow group and BMMF group had a median decrease in hypernasality of two scalar rating points and a similar increase in velar length of approximately 7 mm. A secondary Furlow consistently resolved hypernasality symptoms (80%), but only 56% of patients who underwent BMMF had resolution of hypernasality. The lower percentage of hypernasality resolution in the BMMF group can be attributed to these patients having more severe speech concerns and larger velopharyngeal gaps during speech before surgical management (Fig. 4). Although it was hypothesized that BMMF would result in a greater increase in velar length to treat the larger gap size observed preoperatively, postoperative MRI findings did not support this hypothesis.

Findings from the present study are consistent with the existing literature on palatal lengthening for VPI and expand their findings. In their 2011 study, Mann et al reported that indications to use the BMMF procedure included small velar gaps (<5 mm) and good velar movement,^8^ which is consistent with findings in the present study that patients in the BMMF group saw a median decrease in velopharyngeal gap size of 5 mm. Hens et al reported a mean palate lengthening increase of 7.5 mm, using BMMF as measured on postoperative videofluoroscopy,^30^ which is consistent with findings in the present study that patients in the BMMF group saw a median increase in velar length of 7.5 mm. Substantially expanding on existing knowledge, the present study quantifies changes in velar length after secondary Furlow. We found that secondary Furlow led to a median increase in velar length of 6.9 mm. Most importantly, we found that the degree of velar lengthening achieved was similar for secondary Furlow and BMMF. However, our study did not examine the use of BMMF with patients having small gap sizes, nor did we examine the use of Furlow with patients presenting with large gap sizes.^31^ More research is needed to understand if the anatomic gains made following surgery are seen across all patients or are consistent only when applied using the clinical workflow described in this article.

Although some patients receiving BMMF in the present study gained greater than 7 mm of velar length, many of these patients did not achieve resolution of hypernasality after BMMF. Review of these selected cases revealed that many had limited velar elevation on preoperative imaging, and this persisted on postoperative imaging. This preliminary finding highlights the need for additional research to investigate the role of levator veli palatini muscle contraction as it relates to elevation of the velum.

Because both palate lengthening procedures yielded similar results in the nonsyndromic cleft palate population in the present study, practitioners should consider additional patient and surgeon factors when developing a surgical plan. From the patient’s perspective, a secondary Furlow requires a single surgery, while BMMF may require a second surgery for division and inset of the flap pedicles, depending on surgical technique and individual patient anatomy.^14^ Additionally, some patients may require placement of bite blocks to prevent injury to the BMMF pedicle during the postoperative period.^30^ From a surgeon perspective, it is important for surgeons to use techniques with which they are most experienced, as prior studies in cleft palate surgery show increased complication rates with trials of new techniques.^32^ The cost and burden of care to families should be considered if outcomes are similar within this patient population.

This study has several limitations due to its retrospective nature and all patients being treated at a single hospital. One limitation is the small sample size. In particular, only half of the patients completed a postoperative MRI, so findings from pre- and postoperative MRI comparisons may not represent the entire study population. Further research is needed with a larger sample size to detect differences that may be clinically significant for determining surgical selection. An additional limitation to this study was the bias introduced by the surgical selection pathway, which used gap size from preoperative imaging as a determinant, resulting in patients with larger gap sizes and more severe speech symptoms receiving BMMF. This bias may have influenced results for the change in velopharyngeal gap, as reduction in velopharyngeal gap was limited by the size of the preoperative gap. Finally, while three surgeons performed the operations in these patients, the results may not generalize to surgeons who use substantially different variations of the Furlow or BMMF techniques. Taken together, these limitations suggest caution in generalizing study findings, but do not substantially threaten their validity.

## CONCLUSIONS

Secondary Furlow and buccal myomucosal flaps are effective procedures to treat symptoms of VPI. In the current study, both procedures significantly increased velar length and effective velar length and decreased hypernasality by two scalar points. Anatomical changes persisted 1 year after surgery based on objective measurements with MRI and were associated with improved speech outcomes.

## DISCLOSURES

The authors have no financial interest to declare in relation to the content of this article. This study was supported by the National Institute of Dental & Craniofacial Research of the National Institutes of Health under award numbers K23DE025023 and U01DE029750.

## Supplementary Material


