# Supplementary material for: Comparative Effectiveness of Secondary Furlow and Buccal Myomucosal Flap Lengthening to Treat Velopharyngeal Insufficiency
Source: Plast Reconstr Surg Glob Open. 2023 Nov 3;11(11):e5375. doi: 10.1097/GOX.0000000000005375 (PMC10624468; doi:10.1097/GOX.0000000000005375)
Supplement: Supplementary file 1 [file gox-11-e5375-s001.pdf]

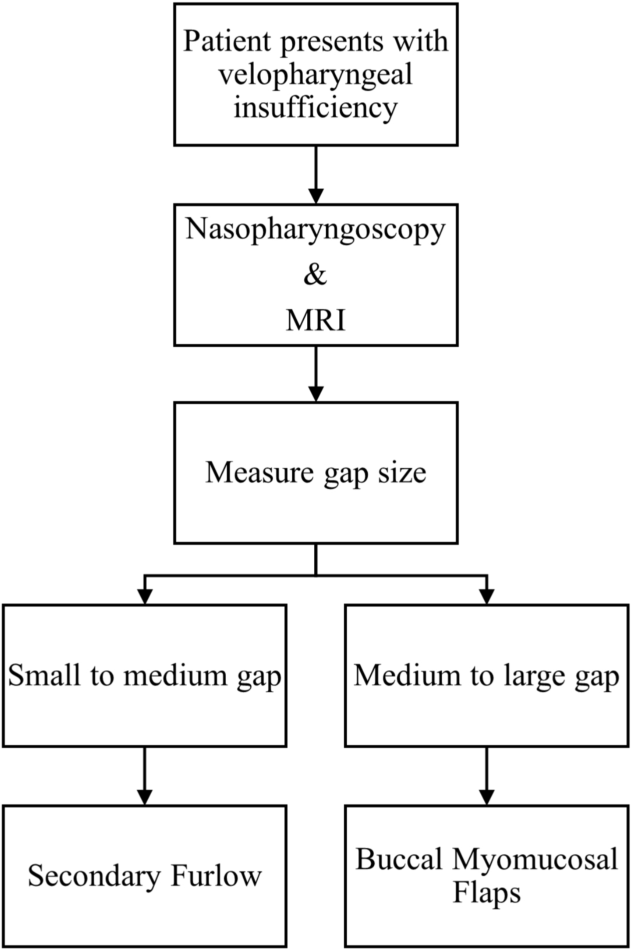

Patient presents with velopharyngeal insufficiency
Nasopharyngoscopy
&
MRI
Measure gap size
Small to medium gap
Medium to large gap
Secondary Furlow
Buccal Myomucosal Flaps
